# Noninvasive continuous versus intermittent arterial pressure monitoring: evaluation of the vascular unloading technique (CNAP device) in the emergency department

**DOI:** 10.1186/1757-7241-22-8

**Published:** 2014-01-29

**Authors:** Julia Y Wagner, Julia S Prantner, Agnes S Meidert, Alexander Hapfelmeier, Roland M Schmid, Bernd Saugel

**Affiliations:** 1 III. Medizinische Klinik, Klinikum rechts der Isar der Technischen Universität München, Ismaninger Strasse 22, 81675 München, Germany; 2 II. Medizinische Klinik und Poliklinik, Klinikum rechts der Isar der Technischen Universität München, Ismaninger Strasse 22, 81675 München, Germany; 3 Institut für Medizinische Statistik und Epidemiologie, Klinikum rechts der Isar der Technischen Universität München, Ismaninger Strasse 22, 81675 München, Germany; 4 Current affiliation: Department of Anesthesiology, Center of Anesthesiology and Intensive Care Medicine, University Medical Center Hamburg-Eppendorf, Martinistrasse 52, 20246 Hamburg, Germany

**Keywords:** Systolic blood pressure, Diastolic blood pressure, Mean arterial pressure, Emergency medicine, Photoplethysmography, Volume clamp method

## Abstract

**Background:**

Monitoring cardiovascular function in acutely ill patients in the emergency department (ED) is of paramount importance. Arterial pressure (AP) is usually monitored using intermittent oscillometric measurements with an upper arm cuff. The vascular unloading technique (VUT) allows continuous noninvasive AP monitoring. In this study, we compare continuous AP measurements obtained by VUT with intermittent oscillometric AP measurements in ED patients. In addition, we aimed to investigate whether continuous noninvasive AP monitoring allows detection of relevant hypotensive episodes that might be missed with intermittent AP monitoring.

**Methods:**

In a German university hospital, 130 ED patients who required AP monitoring were analyzed in this prospective method comparison study. Continuous AP monitoring was performed using VUT (CNAP technology; CNSystems Medizintechnik AG, Graz, Austria) over a 2-hour period. The oscillometric AP values were recorded simultaneously every 15 minutes for the comparison of both methods. For statistical evaluation, Bland-Altman plots accounting for repeated AP measurements per individual were used.

**Results:**

The mean difference (±standard deviation) between AP measurements obtained by VUT and oscillometric AP measurements was -5 mmHg (±22 mmHg) for systolic AP (SAP), -2 mmHg (±15 mmHg) for diastolic AP (DAP), and -6 mmHg (±16 mmHg) for mean AP (MAP), respectively. In the interval between two oscillometric measurements, the VUT device detected hypotensive episodes (≥4 minutes) defined as either SAP <90 mmHg or MAP <65 mmHg in 30 patients and 16 patients, respectively. In 11 (SAP <90 mmHg) and 6 (MAP <65 mmHg) of these patients, hypotension was also detected by the subsequent intermittent oscillometric AP measurement.

**Conclusions:**

VUT using the CNAP system for noninvasive continuous AP measurement shows reasonable agreement with intermittent oscillometric measurements in acutely ill ED patients. Continuous AP monitoring allows immediate recognition of clinically relevant hypotensive episodes, which are missed or only belatedly recognized with intermittent AP measurement.

## Background

Adequate monitoring of cardiovascular function in acutely ill patients is of outstanding importance, as belatedly detected hemodynamic instability might worsen the outcome of these patients [1]. In high dependency unit and intensive care unit (ICU) patients, arterial pressure (AP) is therefore monitored continuously using an arterial catheter placed in the radial or femoral artery. As this invasive method of AP monitoring is associated with risks [2] and requires specially trained medical staff, its application is limited and not suitable for broad use in the emergency department (ED). Patients requiring AP monitoring in the ED are, therefore, routinely connected to automated oscillometric devices which measure AP intermittently at defined time intervals (depending on the patient’s clinical condition). However, oscillometry does not meet the need for continuous AP monitoring. During the last years, methods for continuous noninvasive AP monitoring have become available for evaluation in clinical studies [3-17]. One technology for noninvasive continuous AP assessment is the vascular unloading technique (VUT) [3-11]. The CNAP technology (CNSystems Medizintechnik AG, Graz, Austria) is one of two commercially available AP monitoring systems that use VUT. Previous studies have described its application during general anesthesia and showed clinically acceptable agreement between VUT using the CNAP technology and invasive continuous AP monitoring [5,7,8]. Furthermore, the CNAP technology was evaluated during medical procedures, e.g., interventional endoscopy [18] or spinal anesthesia for caesarean section [19]. The aim of the present study was to compare AP values recorded with VUT (CNAP technology) over a 2-hour period with intermittently measured AP values obtained using oscillometry in acutely ill ED patients. In addition, we aimed to investigate whether continuous noninvasive AP monitoring allows detection of relevant hypotensive episodes that might be missed with intermittent AP monitoring.

## Materials and methods

### Study design, setting, and endpoints

This was a prospective convenience study conducted in the ED of a university hospital (Klinikum rechts der Isar der Technischen Universität München, Munich, Germany). The ethics committee (Ethikkommission der Fakultät für Medizin der Technischen Universität München) approved the study (project number 5745/13). All patients gave written informed consent prior to study enrollment.

The primary endpoint was to compare the accuracy and precision of AP measurements obtained noninvasively and continuously by VUT in comparison with intermittently obtained oscillometric AP measurements (criterion standard).

The secondary endpoint was to evaluate whether continuous AP monitoring using VUT can identify relevant hypotensive episodes that are missed or belatedly detected by intermittent oscillometric measurements.

### Inclusion/exclusion criteria

To be eligible for study enrollment, patients needed to be considered requiring cardiovascular monitoring by the ED medical staff, independently of the present study. Patients with anatomical abnormalities, severe edema of the upper extremities, or a systolic oscillometric AP difference between the right and the left upper arm of more than 10 mmHg were excluded. Critically ill patients requiring an immediate admission to the ICU or emergency procedures (e.g., cardiac catheterization) were also not eligible for study enrollment.

### The CNAP technology used for VUT

The CNAP system used in our study is composed of the CNAP Monitor 500, the CNAP double finger cuff, the CNAP controller, which is attached to the patient’s forearm and connects the finger cuff, and an upper arm cuff for oscillometric AP measurements at the brachial artery used for calibration of the finger cuff-derived values. The CNAP technology is based on the principle of VUT which was originally developed by Peňáz in 1973 [20]. Infrared light is sent through the finger and the transmitted light depends on the absorption by the blood in the finger artery. For AP determination, pressure from the outside finger cuff is applied in order to keep the blood volume in the finger artery constant in accordance to the transmitted light. This corresponding pressure needed to keep the diameter and volume of the finger artery constant throughout the AP cycle correlates with AP [3]. In order to achieve constant volume in the finger arteries, the controller on the forearm makes multiple adjustments per second. The systolic AP (SAP) and diastolic AP (DAP) values measured with VUT are calibrated to the values obtained by oscillometric upper arm cuff measurements using a proprietary transfer function and mean AP (MAP) is adjusted accordingly.

### Study measurements

Patients who were already routinely connected to the ED standard monitoring device (Infinity® Delta Monitor, Dräger Medical Deutschland GmbH, Lübeck, Germany) by the ED medical staff were then additionally AP monitored by VUT using the CNAP technology installed by a research assistant. For this purpose, the CNAP oscillometric upper arm cuff for calibration and the CNAP finger cuff were attached on one arm of the patient whereas the other arm was used for AP measurements with the brachial cuff connected to the standard ED monitor. The appropriate size both for the CNAP upper arm cuff and for the finger cuff (small, medium or large) was chosen by the research assistant depending on the upper arm and finger circumferences.

Intermittent oscillometric AP measurements were performed at the beginning of a measurement and every 15 minutes for a 2-hour-period. The research assistant marked the moment of appearance of the oscillometric AP values on the patient standard monitor’s display by putting an event in the data recording of the CNAP monitor. AP measurement by the CNAP upper arm cuff for calibration was performed according to the manufacturer’s recommendation (every 30 minutes). For both methods, SAP, DAP, and MAP values were recorded. Therapeutic decisions were based solely on the oscillometric AP measurements used in the clinical routine.

### Patients

After checking inclusion and exclusion criteria, 150 patients who received medical treatment in the ED were enrolled in this study over a time period of 3 months. The fact that only one trained research assistant performed all study measurements explains the relatively small number of study patients considering that over 2000 patients actually presented to the ED during the study period.

Four patients were excluded because of excessive movement of the study limb, 9 were excluded because of premature interruption of measurement for organizational reasons, and in 7 patients technical problems occurred either with oscillometric or continuous noninvasive measurements. Data sets of 130 patients were available for the final statistical analyses. The patients’ characteristics and the reasons for admission to the ED are detailed in Table 1.

**Table 1:** Patients’ characteristics and reason for emergency department admission

**Patients’ characteristics**	
Sex (male), n (%)	69 (53)
Age, years	66 (51–74)
Height, cm	170 (164–176)
Body weight, kg	73 (65–85)
**Reason for emergency department treatment**	**N (%)**
Syncope	17 (13)
Gastrointestinal bleeding	16 (12)
Pneumonia	10 (8)
Hypertensive urgency/emergency	9 (7)
Acute renal failure	8 (6)
Chronic obstructive pulmonary disease/Asthma	7 (5)
Acute coronary syndrome	6 (5)
Congestive heart failure	6 (5)
Infection	6 (5)
Systemic inflammatory response syndrome/Sepsis	6 (5)
Subdural hematoma	6 (5)
Pulmonary embolism	5 (4)
Severe pancreatitis	5 (4)
Others	23 (18)

Data are presented as absolute and percentage frequencies or as median and interquartile range for continuous parameters. Percentages may not total 100 due to rounding.

### Data processing and statistical analysis

To evaluate the accuracy and precision of VUT we calculated the mean of the differences (=bias), standard deviation, and limits of agreement (1.96 × standard deviation). The differences between VUT-derived and oscillometric AP measurements were calculated by subtracting from the VUT-derived values the oscillometric AP values. We averaged the obtained VUT measurements over a period of 30 seconds before the corresponding oscillometric AP value was recorded and compared these averaged VUT-derived AP values with the respective oscillometrically obtained values. As the oscillometric AP values were recorded at the start of a 2-hour-measurement period and every 15 minutes, 9 paired measurements for each SAP, DAP, and MAP were available for AP comparison per individual. Before further data processing, VUT-derived waveforms were visually inspected and apparent marked outliers, basically due to excessive study limb movement, were excluded. The percentage of excluded continuous AP recordings was 1.96% of all measurements obtained by VUT.

For patient characteristics and the duration of hypotensive episodes, we calculated the median and interquartile ranges (i.e., 25% to 75% percentile range). For AP values assessed either by intermittent oscillometric measurements or by VUT, mean ± standard deviation were calculated. The AP values obtained by both methods were compared using the Bland-Altman method accounting for repeated measurements per individual [21]. The relationship between differences between the two methods and mean measurements was tested by mixed model regression. Furthermore, we identified hypotensive episodes recorded by VUT in the interval between two oscillometric measurements. A hypotensive episode was defined as a SAP <90 mmHg or MAP <65 mmHg over a consecutive time period of at least 4 minutes length.

Statistical data analysis was performed using IBM SPSS Statistics 21 (SPSS Inc., Chicago, IL, USA) and the statistical software package R (The R Foundation for Statistical Computing, Vienna, Austria).

## Results

**Figure 1:**
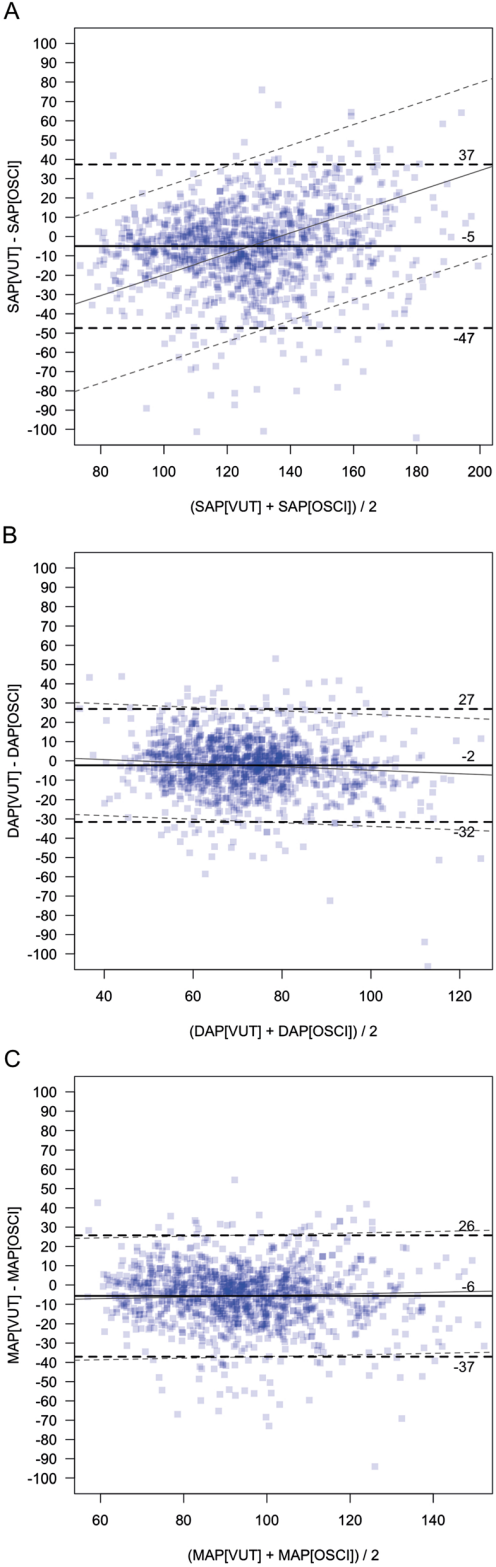
**Comparison between intermittent oscillometric and continuous noninvasive arterial pressure measurements.** Bland-Altman plots accounting for repeated measurements for the comparison of arterial pressure measurements using the vascular unloading technique (VUT) with arterial pressure measurements using oscillometry (OSCI) are presented. Data are separately shown for systolic arterial pressure (SAP-VUT vs. SAP-OSCI) **(A)**, diastolic arterial pressure (DAP-VUT vs. DAP-OSCI) **(B)**, and mean arterial pressure (MAP-VUT vs. MAP-OSCI) **(C)**. The bias is illustrated by a continuous horizontal line. The dashed horizontal lines represent the 95% limits of agreement, i.e., bias ± 1.96 * standard deviation. Diagonal lines indicate the non-uniform relation between differences and mean values assessed by mixed model regression.

For the evaluation of agreement between the noninvasive continuous AP measurements using VUT and the intermittently obtained oscillometric AP measurements, 1,155 paired AP measurements in 130 patients were statistically analyzed. The mean value (±standard deviation) of VUT-derived AP was 125 mmHg (±27 mmHg) for SAP, 71 mmHg (±15 mmHg) for DAP, and 92 mmHg (±19 mmHg) for MAP, respectively (Table 2). The mean value (±standard deviation) of AP obtained by the oscillometric method was 130 (±24 mmHg), 74 (±17 mmHg), and 97 mmHg (±20 mmHg) for SAP, DAP, and MAP, respectively. The mean difference (±standard deviation; 95% limits of agreement) between AP measurements obtained by VUT and oscillometric AP measurements was -5 mmHg (±22; -47 to +37 mmHg) for SAP, -2 mmHg (±15; -32 to +27 mmHg) for DAP, and -6 mmHg (±16; -37 to +26 mmHg) for MAP, respectively. Figure 1 shows the corresponding Bland-Altman plots for SAP, DAP, and MAP.

**Table 2:** Overview of the arterial pressure variables measured by the oscillometric method and vascular unloading technique and the differences between the two methods

	**Mean value OSCI (± SD)**	**Mean value VUT (± SD)**	**Mean difference (± SD; LOA)**
**SAP**	130 (± 24)	125 (± 27)	-5 (± 22; -47 to + 37)
**DAP**	74 (± 17)	71 (± 15)	-2 (± 15; -32 to + 27)
**MAP**	97 (± 20)	92 (± 19)	-6 (± 16; -37 to + 26)

All arterial pressure values are given in mmHg. OSCI, oscillometric arterial pressure measurement; SD, standard deviation; SAP, systolic arterial pressure; DAP, diastolic arterial pressure; MAP, mean arterial pressure; VUT, vascular unloading technique; LOA, limits of agreement.

In the interval between two oscillometric measurements, VUT detected hypotensive episodes (≥4 minutes) defined as either SAP <90 mmHg or MAP <65 mmHg at least once in 30 patients (with a total of 67 episodes) and 16 patients (with a total of 27 episodes), respectively. The median duration of these episodes was 6.7 (4.5 – 12.1) and 5.7 (4.8 – 8.7) minutes, respectively. In 3 (SAP <90 mmHg) and 2 (MAP <65 mmHg) measurements in 2 and 1 patients, respectively, the recorded hypotensive episodes lasted at least 45 minutes. In 11 (SAP <90 mmHg) and 6 (MAP <65 mmHg) of the patients (i.e., in 25 and 7 of the episodes, respectively), hypotension was also detected by the subsequent intermittent oscillometric AP measurement. As the time interval between two oscillometric measurements was 15 minutes, the detection of a hypotensive episode by VUT took place with an average time advantage of 8.1 minutes for MAP <65 mmHg and 9.5 minutes for SAP <90 mmHg compared to the subsequent oscillometric measurement.

## Discussion

This exploratory study compares continuous noninvasive AP monitoring obtained by VUT (CNAP technology) with the method of intermittent oscillometric AP measurements which is widely used and generally accepted by health care professionals. Thus, the oscillometric method is also routinely used and considered as criterion standard for AP monitoring in acutely ill patients presenting to the ED. One major limitation of this diagnostic device is, however, that it does not provide uninterrupted monitoring of the patient’s AP behavior. Particularly in hemodynamically unstable patients, only continuous AP recordings including real-time visualization of the AP waveform allow the treating ED staff to rapidly identify hypotensive phases and treat the patient accordingly in order to avoid permanent organ damage due to hypoperfusion. In clinical routine, the criterion standard for continuous AP monitoring are arterial catheter-derived AP measurements usually reserved for ICU patients as invasive AP measurements are often not practically applicable in the ED. However, in specific medical conditions, the first hours that a patient often spends in the ED when admitted to the hospital might be crucial when it comes to physical long-term damage and survival. Kumar and colleagues demonstrated the importance of early detection of hypotension in septic shock patients, as the administration of antimicrobial therapy within the first hour of recorded hypotension was associated with a significantly higher survival rate [1]. Given the possible damage caused by unregistered episodes of hypotension, but also the limitations in establishing invasive AP monitoring in the ED, a continuous noninvasive AP monitoring technology like the CNAP system might help to increase patient safety during the first hours of medical treatment. There already exist validation studies comparing CNAP-derived AP with invasively assessed AP measurements that reported clinically acceptable agreement for SAP, DAP, and MAP and, therefore, suggested CNAP as an alternative for invasive AP monitoring [5,8]. Furthermore, recent studies were able to demonstrate the potential value of continuous noninvasive AP monitoring by VUT using the CNAP device compared with intermittent oscillometric AP monitoring: Ilies and colleagues detected more hypotensive episodes after spinal anesthesia in women requiring caesarean section using the CNAP device than with the intermittent AP measurements [19]. In a study by Siebig and colleagues, rapid AP changes during sedation in interventional endoscopy patients were identified using VUT for continuous AP monitoring which were not detected by the standard oscillometric method [18].

Nevertheless, the comparability of the noninvasive device with the current criterion standard is important for its use in clinical practice. Our method comparison study revealed a satisfactory agreement between noninvasive AP monitoring using VUT and intermittent oscillometric AP measurements resulting in a bias (95% limits of agreement) of -5 mmHg (-47 to +37 mmHg) for SAP, -2 mmHg (-32 to +27 mmHg) for DAP, and -6 mmHg (-37 to +26 mmHg) for MAP, respectively.

In a similar study, Nowak and colleagues compared intermittent oscillometric AP measurements with noninvasive continuous AP measurements using the Nexfin device (BMEYE, Amsterdam, The Netherlands) that also uses VUT in 40 patients treated in the ED [22]. In this trial, a mean difference and 95% limits of agreement of +0.87 mmHg and -45.8 to +47.54 mmHg for SAP, of -1.24 mmHg and -31.74 to +29.27 mmHg for DAP, and of -2.05 mmHg and -33.83 to +29.74 mmHg for MAP were observed. The respective bias for SAP, DAP, and MAP in the study of Nowak and colleagues is lower indicating a higher accuracy of the noninvasive device compared with our results, whereas the limits of agreement and therefore the precision are reasonably comparable. Among other things, these differences may be explainable by the fact that the CNAP device uses automated calibration to upper arm oscillometric AP whereas the Nexfin device uses a different technique [10]. To what extent the calibration method has an impact on the accuracy and precision of VUT is the subject of further studies. Regarding the types of diagnoses, the patients investigated in the study of Nowak et al. were comparable with those included in our trial with a smaller total number of cases enrolled in this previous study as compared with our work. In contrast to our study, the exclusion criteria of Nowak and colleagues did not take into account AP differences between the right and left upper arm which might have had an impact on study results as they also used one patient’s upper extremity for oscillometric measurements and the contralateral one for continuous noninvasive monitoring.

In addition to the sole comparison of methods, we analyzed AP recordings of each patient for hypotensive phases in the interval between two oscillometric measurements. For this purpose we deliberately chose 4 minutes as cut-off value in order to ensure that only clinically relevant hypotensive phases are identified.

Several limitations of our study have to be mentioned to ensure the correct interpretation of our results. Firstly, we did not define the limits of agreement a priori. Although relevant guidelines for the comparison of two AP measurement methods from the Association for the Advancement of Medical Instrumentation (AAMI) exist, these guidelines explicitly do not cover finger AP measurement devices and no other guidelines addressing this type of measurement comparison studies are available [23]. Considering this fact, it would have been arbitrary to define limits of agreement a priori. Secondly, critically ill patients who presented hemodynamically unstable were immediately admitted to the ICU, provided that an ICU bed was available. Therefore, patients included in our study were relatively hemodynamically stable.

According to our study, noninvasive continuous AP assessment using VUT might be a promising monitoring option for acutely ill ED patients with the potential for increasing patient safety. An application of the noninvasive continuous AP monitoring technique in the ED is conceivable because devices using VUT are easy to apply and safer in contrast to invasive AP measurements. Therefore it seems to be particularly suitable in the ED as in many patients there is no clear indication for invasive AP monitoring or time until invasive AP measurements become possible has to be bridged.

## Conclusions

In conclusion, VUT using the CNAP system for noninvasive continuous AP measurement shows reasonable agreement with intermittent oscillometric measurements in acutely ill ED patients. Continuous AP monitoring allows immediate recognition of clinically relevant hypotensive episodes, which are missed or only belatedly recognized with intermittent AP measurement.

## Competing interests

JYW, ASM, and BS received refunds of travel expenses from CNSystems Medizintechnik AG (Graz, Austria). All other authors have no conflict of interest to disclose. CNSystems Medizintechnik AG (Graz, Austria) provided the technical equipment needed for this study. The company was not involved in the study design, in the collection, analysis, and interpretation of data, writing of the manuscript, and in the decision to submit the manuscript for publication.

## Authors’ contributions

JYW and BS were responsible for study design and conception, patient recruitment, data collection, analysis and interpretation of data, and drafted the manuscript. JSP carried out patient recruitment, data collection, and revised the article for important intellectual content. ASM participated in the development of study design and conception and revised the article for important intellectual content. AH participated in the analysis and interpretation of data and the draft of the manuscript. RMS participated in study design and conception and revised the article for important intellectual content. All authors read and approved the final manuscript.
